# Is Social Capital a Determinant of Oral Health among Older Adults? Findings from the English Longitudinal Study of Ageing

**DOI:** 10.1371/journal.pone.0125557

**Published:** 2015-05-18

**Authors:** Patrick Rouxel, Georgios Tsakos, Panayotes Demakakos, Paola Zaninotto, Tarani Chandola, Richard Geddie Watt

**Affiliations:** 1 UCL Research Department of Epidemiology and Public Health, University College London, London, United Kingdom; 2 Cathie Marsh Institute for Social Research (CMIST), School of Social Sciences, University of Manchester, Manchester, United Kingdom; UNC School of Dentistry, University of North Carolina-Chapel Hill, UNITED STATES

## Abstract

There are a number of studies linking social capital to oral health among older adults, although the evidence base mainly relies on cross-sectional study designs. The possibility of reverse causality is seldom discussed, even though oral health problems could potentially lead to lower social participation. Furthermore, few studies clearly distinguish between the effects of different dimensions of social capital on oral health. The objective of the study was to examine the longitudinal associations between individual social capital and oral health among older adults. We analyzed longitudinal data from the 3^rd^ and 5^th^ waves of the English Longitudinal Study of Ageing (ELSA). Structural social capital was operationalized using measures of social participation, and volunteering. Number of close ties and perceived emotional support comprised the functional dimension of social capital. Oral health measures were having no natural teeth (edentate vs. dentate), self-rated oral health and oral health-related quality of life. Time-lag and autoregressive models were used to explore the longitudinal associations between social capital and oral health. We imputed all missing data, using multivariate imputation by chained equations. We found evidence of bi-directional longitudinal associations between self-rated oral health, volunteering and functional social capital. Functional social capital was a strong predictor of change in oral health-related quality of life – the adjusted odds ratio of reporting poor oral health-related quality of life was 1.75 (1.33–2.30) for older adults with low vs. high social support. However in the reverse direction, poor oral health-related quality of life was not associated with changes in social capital. This suggests that oral health may not be a determinant of social capital. In conclusion, social capital may be a determinant of subjective oral health among older adults rather than edentulousness, despite many cross-sectional studies on the latter.

## Introduction

There is an increasing number of studies that suggest social capital may have a beneficial influence on oral health, although most do not distinguish between two key aspects of social capital—the structural and functional dimensions [1]. The distinction between the different forms and dimensions of social capital is important because their associations with health may vary [2,3]. The structural dimension emphasizes the behavioral aspects of the concept, namely participation in social activities and voluntary organizations. The functional dimension refers to people’s subjective values and perceptions, and emphasizes the relational content and quality of social interactions within the structure of social relationships [2].

Structural social capital is shaped by institutions, policies, and culture [4], which potentially have strong influences on objective population health indicators. A recent meta-analysis of cohort studies showed an inverse association between structural social capital—in terms of social participation and social networks—and mortality. More extensive social networks and greater social participation were linked to lower risk of mortality [5]. Volunteering is a core component of structural social capital. A review of the evidence from experimental and cohort studies on volunteering and health concluded that there was a strong association between volunteering and decreased mortality from cohort studies, although associations with better mental well-being from observational studies were not confirmed by experimental studies [6]. On the other hand, perceived social support, a measure of functional social capital, showed no association with mortality [5,7]. Functional social capital is believed to shape attitudes and values to behavior [4], and appears to be strongly related to mental health in particular [8].

Results from different studies on social capital and health are difficult to compare because of the lack of consistency and the multiple ways of conceptualizing, operationalizing and measuring social capital [9]. This is particularly true for studies on social capital and oral health among older adults. Some studies found associations between structural dimensions of social capital, such as participation in political and social groups, and clinical measures of oral health, such as number of remaining teeth [10–12]. However, in other studies of older Japanese adults, volunteering was not associated with the number of teeth [11,13]. One study revealed that the frequency of contact with friends and relatives was not associated with periodontitis (gum disease) in US male health professionals [14].

Differential associations have also been reported between functional social capital and clinical measures of oral health [11,15,16]. Social support was not associated with the number of remaining teeth among older Japanese adults [11]. Older adults in the US with fewer close friends had higher rates of decayed teeth and root decay, but there was no association with edentulism (no natural teeth) [15]. The number of close friends and need for social support were not associated with periodontal disease in the same US population [16]. In contrast, Merchant et al. [14] showed that US men with more social support were less likely to develop periodontitis. Differential results have also been reported between functional social capital and subjective measures of oral health [15,17]. Higher levels of social support, measured by the number of close friends, were associated with better self-rated oral health among edentate older adults in the US, but not among the dentate [17]. In the same NHANES sample, need for emotional social support was associated with poorer self-rated oral health [15].

Most of these studies reported significant associations between some aspects of social capital and oral health. However, only one study [14] used longitudinal data but this was conducted only amongst a selected sample of medical professionals. This limits our ability to understand the association between social capital and oral health amongst older adults. A related issue is to establish the direction of the association between social capital and oral health. Many studies assumed a unidirectional association from social capital to oral health. It has been claimed that low social capital has negative effects on oral health status [12], though it may also be reasonable to consider a reverse direction for that association, whereby individuals with poor oral health are less able to participate in social activities or volunteer. Moreover, social capital and oral health may influence each other. The debate on this issue is still ongoing, as none of the studies have explicitly addressed this question.

This study explored the bi-directional longitudinal associations between structural and functional dimensions of social capital and oral health in a large national sample of older adults in England. The research questions were: 1) whether social capital predicted changes in oral health, and 2) whether oral health predicted changes in social capital, among older adults.

## Materials and Methods

### Study Population

Data from the 3^rd^ and 5^th^ waves of the English Longitudinal Study of Ageing (ELSA) were analyzed. ELSA is a prospective observational study of community-dwelling people aged 50 years and over in England. Participants were recruited from households that had participated in the Health Survey for England in 1998, 1999, and 2001. At the first wave, in 2002–03, the sample comprised 11,391 core members and was designed to be nationally representative. Follow-up interviews took place every two years but oral health status was only assessed at wave 3 (2006–07) and wave 5 (2010–11). So, for the purposes of this study, baseline refers to wave 3 and follow-up to wave 5. Data were collected using Computer Assisting Personal Interview (CAPI) and the self-completion questionnaire. Participants who were living in institutions or who had proxy respondents were excluded from the study population. Only participants who completed the interviews in person were eligible for the analysis. Ethical approval for all the ELSA waves was granted from NHS Research Ethics Committees under the National Research and Ethics Service (NRES) and all participants have given written informed consent. For further information see here: http://www.nres.nhs.uk/. The technical details on sampling and all related documentation can be found at: http://www.ifs.org.uk/elsa/.

### Assessment of oral health

Edentulousness was measured through self-assessment of the presence of natural teeth. Respondents were asked: ‘in relation to dental health, which of the following applies to you?’ choosing from four categories: “no natural teeth and wear denture”; “both natural teeth and denture(s)”; “only natural teeth”; “neither natural teeth nor dentures”. A dichotomized variable was derived: dentate (having some natural teeth) versus edentate (not having any). Edentulousness is irreversible, so it is a robust and objective measure of total tooth mortality [18].

Self-rated oral health is a broad multidimensional subjective assessment of oral health. It reflects current oral health status, but it could also tap on the mood and emotional state of the respondent [19]. Self-rated oral health has been shown to be a valid, reliable and cost-effective tool to measure oral health [20]. Answers to the question “Would you say dental health (mouth, teeth and/or dentures) is…”: were dichotomized into ‘good’ (excellent/very good/good) and ‘poor’ (fair/poor).

Oral health-related quality of life was measured using a simplified version of the Oral Impacts on Daily Performances (OIDP) questionnaire for elderly populations [21]. This simplified version consists of five daily performances. Respondents were asked if they had experienced any difficulties in eating food, speaking, smiling, or had problems with emotional stability or socializing, due to problems with teeth, mouth or dentures in the past six months. A dichotomized variable was derived distinguishing between participants reporting at least one oral impact against those reporting none.

### Assessment of social capital

The ELSA questions on social capital form part of the harmonized set of items developed and validated by the Office for National Statistics (ONS) [22]. Structural social capital was measured using questions on volunteering and membership of organizations. Volunteering was assessed in the CAPI questionnaire with respondents reporting the frequency they engaged in voluntary work, choosing from ‘twice a month or more’ to ‘never’. Those who reported volunteering once a year or more were classified as volunteers. The question on membership in organizations was included in the self-completion questionnaire. Participants were asked to indicate whether they were a member of any organization, club or society and how many committee meetings they attended in a year. A variable on membership status with three categories was derived: ‘active member’ (attending at least one meeting in a year), ‘passive member’ (member of at least one organization but did not attend any committee meeting in a year), and ‘not a member’.

Functional social capital was measured using questions on the number of close ties and perceived emotional social support. The number of close ties was assessed by summing up separate responses on close ties to children, relatives and friends on the following question: ‘how many of your (children/relatives/friends) would you say you have a close relationship with?’ In addition, ELSA measures emotional closeness between the respondent and their partner using the following question: ‘how close is your relationship with your spouse or partner?’ Respondents who characterized their relationship with their spouse/partner as ‘very close’ or ‘quite close’ were included in the measure of number of close ties. The derived score was positively skewed and hence grouped into tertiles. Respondents in the highest tertile had 10 or more close ties. Those in the lowest tertile of close ties had a range from 0 to 6 close ties.

Perceived emotional social support was measured using a 3-item scale that covers empathy, dependability and confiding. Participants were asked about emotional support perceived from spouse, children, other relatives, and friends. The four possible answers were not at all (0), a little (1), some (2), a lot (3). Items scores were summed to obtain a social support scale for all types of relationship combined ranging from 0 (absolute lack of social support from all sources) to 36 (highest possible score). The derived social support scale was negatively skewed and hence grouped into tertiles. Respondents in the highest tertile of social support scored between 28–36 whereas those in the lowest tertile had a score that ranged from 0–21.

### Covariates at baseline

Age was coded into three groups to reflect different stages of life: 50–64 years (when most respondents are still working); 65–74 years (when most respondents have retired but are still fairly active); and 75 years and older (when most respondents have health problems and need family and/or community support). Other covariates included gender, cohabiting status (cohabiting or not with a partner), educational qualifications (some vs. no qualifications) and labor market status (in paid employment, retired, and ‘other’ that included unemployed/permanently sick or disabled/looking after family). Household wealth quintiles were used as a measure of socio-economic position, as this has been shown to best capture the material resources available to older adults and be the best socio-economic predictor of health in ELSA [23]. Wealth was calculated net of debt and included the total value of any housing, financial and physical wealth owned by the household. Health and behavioral covariates included self-rated general health (very good/good vs. fair/bad/very bad), limiting long-standing illness, a measure of depression using the 8-item version of Centre for Epidemiological Studies Depression Scale (CES-D) in which participants reporting 4 or more depressive symptoms were classified as being depressed [24], and smoking status (never smoked, ex-smoker, and current smoker). It is particularly important to control for socio-economic and health factors as these may confound the association between social capital and oral health.

### Missing data and multiple imputation

Missing data and attrition is an important concern for longitudinal studies, especially those on older adults. If attrition is systematically related to outcomes of interest or to variables correlated with these outcomes, the study may no longer be representative of the population. Furthermore, any estimated associations may also be biased [25].

A flow chart of the different processes resulting in missing data from wave 3 to wave 5 is discussed and displayed in Text A and Fig A in S1 File. The cumulative effect of missing data in several variables leads to exclusion of a substantial proportion of the original sample (N = 8,552), which resulted in the longitudinal complete case sample of 3,519 participants. Participants who were more likely not to be included in the longitudinal complete case sample were aged 65 or older; male; not living with a partner; with no educational qualifications; in a lower socio-economic position; with poor self-rated general and oral health, current smokers; and with low social capital (Table A in S1 File).

In order to reduce potential biases arising from missing data, a multiple imputation model using chained equations implemented in STATA [26,27] was carried out to create 50 imputed datasets [28,29]. Further details of the multiple imputation process are described in Text B and Table B in S1 File.

### Statistical analysis

Preliminary analyses described the data, presenting first the prevalence (%) of all baseline variables for complete case and imputed data. Secondly, prevalence of outcomes at follow-up by baseline predictors and covariates were presented for imputed data and the F-test was used to determine the statistical significance of the associations. To assess the longitudinal associations between social capital at baseline and subsequent oral health, time-lag models were fitted as follows: the social capital predictor variable at baseline were related to the oral health outcomes at follow-up, age-adjusted (Model 1) and sequentially adjusted for baseline demographic, socio-economic, general health and smoking status (Model 2). Time-lag models take into account the temporal sequence of a possible cause and effect. Autoregressive models were then fitted by adjusting for the baseline dependent variable (Model 3). Autoregressive models help to “remove” the cross-sectional part of the relationships, in order to estimate the real influence of the predictor variables on the outcome variables [30]. The autoregressive models, thus examined the association of the baseline predictors with change in the outcome between waves 3 and 5.

Similarly, as described above, the reverse temporal association between oral health at baseline predicting social capital at follow-up was examined. For binary outcomes, we used logistic regression to estimate Odds Ratios and 95% Confidence Intervals (OR, 95%CI) for the association between exposure and outcome. For categorical outcomes, we used multinomial logistic regression to estimate the Relative Risk Ratios and 95% Confidence Intervals (RRR, 95%CI). In order to simplify the tables, only one set of RRRs for the extreme categories of social capital was presented in each of the three models. We used the Wald statistic to calculate 95% confidence intervals and p-values. Interaction effects between social capital and the demographic, socio-economic and depression variables were tested for in the fully adjusted regression models.

The results using the imputed dataset are presented in the main text. In order to limit the number of tables in the text, we summarized the results of models 2 and 3, with the detailed model results discussed and presented in Text C and Tables C-I in S1 File. The results for the complete case dataset are summarized in Tables J and K in S1 File.

All analyses were performed using Stata/SE 12.1 (StataCorp LP)

## Results

### Descriptive findings

The distribution of all the baseline variables in the complete case (N = 3,519) and imputed (N = 7,899) datasets are displayed in Tables 1 and 2. Differences between the two datasets were found with the complete case dataset being younger, more educated, wealthier, healthier (both general and oral health), and more actively engaged in social activities compared to the imputed dataset.

**Table 1:** Distribution % (n) of the characteristics of the ELSA sample at baseline (2006–07) by oral health outcomes at follow-up (2010–11).

Covariates at baseline (2006–07)			Oral health status at follow-up (2010–11)		
	Complete case	Imputed	Poor self-rated oral health	Edentate	OIDP^1^
	N = 3,519	N = 7,899	n = 1,408^a^	n = 1,249^b^	n = 826^c^
**Age group (years)**					
50–64	60.4% (2,127)	54.6% (4,316)	18.8% (811)	6.8% (294)	10.4% (449)
65–74	26.9% (946)	26.6% (2,104)	17.1% (360)	19.8% (416)	10.9% (229)
75+	12.7% (446)	18.7% (1,479)	16.0% (237)	36.4% (539)	10.0% (148)
p-value*			0.067	<0.001	0.724
**Gender**					
Men	42.9% (1,509)	43.9% (3,471)	19.6% (680)	13.4% (467)	10.8% (376)
Women	57.1% (2,010)	56.1% (4,428)	16.4% (728)	17.7% (782)	10.2% (450)
p-value*			0.001	<0.001	0.413
**Cohabiting status**					
Living with partner	72.9% (2,566)	69.5% (5,491)	16.5% (908)	12.2% (668)	9.5% (520)
Not living with partner	27.1% (953)	30.5% (2,408)	20.8% (500)	24.1% (681)	12.7% (306)
p-value*			<0.001	<0.001	<0.001
**Educational qualifications**					
Some qualifications	80.6% (2,837)	71.2% (5,622)	16.4% (923)	10.2% (574)	9.7% (546)
No qualification	19.4% (682)	28.8% (2,277)	21.3% (485)	29.7% (675)	12.3% (280)
p-value*			<0.001	<0.001	0.005
L**abour market status**					
In paid employment	40.6% (1,428)	37.0% (2,923)	16.8% (492)	5.6% (163)	9.3% (271)
Retired	46.2% (1,626)	48.4% (3,824)	16.8% (635)	23.5% (898)	10.5% (402)
Other	13.2% (465)	14.6% (1,152)	24.8% (281)	16.3% (188)	13.3% (153)
p-value*			<0.001	<0.001	0.003
**Wealth quintile**					
Wealthiest quintile	27.9% (980)	22.3% (1,765)	13.6% (241)	5.6% (98)	8.7% (153)
4th	23.7% (834)	20.7% (1,639)	15.2% (250)	9.9% (162)	7.2% (119)
3rd	19.2% (677)	20.4% (1,609)	17.8% (287)	16.3% (262)	10.0% (161)
2nd	17.3% (610)	19.4% (1,529)	19.5% (298)	20.7% (316)	12.5% (191)
Poorest quintile	11.9% (418)	17.2% (1,357)	24.5% (332)	30.3% (411)	14.9% (202)
p-value*			<0.001	<0.001	<0.001
**Self-rated general health**					
Good	75.9% (2,670)	70.0% (5,527)	13.0% (716)	12.1% (669)	7.8% (432)
Poor	24.1% (849)	30.0% (2,372)	29.1% (692)	24.4% (580)	16.6% (394)
p-value*			<0.001	<0.001	<0.001
**Limiting long-standing illness**					
No	71.9% (2,531)	68.1% (5,379)	14.5% (780)	12.6% (677)	8.3% (448)
Yes	28.1% (988)	31.9% (2,520)	24.9% (628)	22.7% (572)	15.0% (378)
p-value*			<0.001	<0.001	<0.001
**Depression (≥4 symptoms)**					
No	84.5% (2,975)	80.3% (6,342)	14.8% (939)	14.6% (929)	8.8% (559)
Yes	15.5% (544)	19.7% (1,557)	30.1% (469)	20.5% (320)	17.2% (267)
p-value*			<0.001	<0.001	<0.001
**Smoking status**					
Never-smoked	41.2% (1,451)	38.8% (3,064)	13.6% (418)	10.7% (327)	7.0% (214)
Ex-smoker	46.4% (1,632)	46.4% (3,668)	18.0% (661)	17.6% (644)	11.3% (416)
Current smoker	12.4% (436)	14.8% (1,167)	28.2% (329)	23.8% (278)	16.8% (196)
p-value*			<0.001	<0.001	<0.001
**Membership status**					
Active member	38.9% (1,369)	32.9% (2,600)	15.1% (394)	11.0% (286)	10.3% (268)
Passive member	37.3% (1,323)	38.1% (3,013)	17.2% (520)	14.5% (436)	9.6% (290)
Not a member	23.5% (827)	29.0% (2,286)	21.6% (494)	23.0% (527)	11.7% (268)
p-value*			<0.001	<0.001	0.122
**Volunteering status**					
Volunteering	34.0% (1,198)	27.4% (2,166)	13.4% (290)	10.1% (218)	9.6% (208)
Not volunteering	66.0% (2,321)	72.6% (5,733)	19.5% (1,118)	18.0% (1,031)	10.8% (618)
p-value*			<0.001	<0.001	0.18
**Number of close ties**					
Highest tertile	33.1% (1,166)	33.4% (2,637)	15.1% (399)	15.3% (402)	8.3% (218)
Middle tertile	31.1% (1,095)	28.0% (2,210)	16.9% (374)	13.8% (305)	10.0% (222)
Lowest tertile	35.7% (1,258)	38.6% (3,052)	20.8% (635)	17.7% (542)	12.6% (386)
p-value*			<0.001	0.003	<0.001
**Social support**					
Highest tertile	31.1% (1,093)	29.8% (2,357)	14.1% (332)	11.9% (281)	6.9% (163)
Middle tertile	33.7% (1,187)	36.9% (2,915)	16.4% (478)	15.4% (448)	10.1% (295)
Lowest tertile	35.2% (1,239)	33.3% (2,627)	22.7% (598)	19.8% (520)	14.0% (368)
p-value*			<0.001	<0.001	<0.001

^1^Oral Impacts on Daily Performances

^a^Imputed n (complete case n = 566)

^b^Imputed n (complete case n = 393)

^c^Imputed n (complete case n = 337)

^*^F-test statistics for the p-value

**Table 2:** Distribution % (n) of the characteristics of the ELSA sample at baseline (2006–07) by social capital outcomes at follow-up (2010–11).

Covariates at baseline (2006–07)			Social capital at follow-up (2010–11)			
	Complete case	Imputed	Not a member of any organisations	Not volunteering	Lowest tertile of close ties	Lowest tertile of social support
	N = 3,519	N = 7,899	n = 2,432^a^	n = 5,752^b^	n = 3,075^c^	n = 2,773^d^
**Age group (years)**						
50–64	60.4% (2,127)	54.6% (4,316)	30.6% (1,323)	70.6% (3,048)	38.7% (1,672)	30.8% (1,329)
65–74	26.9% (946)	26.6% (2,104)	28.1% (591)	69.3% (1,458)	36.6% (770)	34.3% (723)
75+	12.7% (446)	18.7% (1,479)	35.0% (518)	84.2% (1,246)	42.8% (633)	48.7% (721)
*p-value* ^*^			<0.001	<0.001	0.002	<0.001
**Gender**						
Men	42.9% (1,509)	43.9% (3,471)	28.3% (984)	73.6% (2,553)	40.8% (1,418)	35.5% (1,232)
Women	57.1% (2,010)	56.1% (4,428)	32.7% (1,448)	72.2% (3,199)	37.4% (1,657)	34.8% (1,541)
*p-value* ^*^			<0.001	0.209	0.007	0.334
**Cohabiting status**						
Living with partner	72.9% (2,566)	69.5% (5,491)	28.8% (1,581)	70.2% (3,856)	34.9% (1,916)	21.6% (1,184)
Not living with partner	27.1% (953)	30.5% (2,408)	35.3% (851)	78.7% (1,896)	48.1% (1,159)	66.0% (1,589)
*p-value* ^*^			<0.001	<0.001	<0.001	<0.001
**Educational qualifications**						
Some qualifications	80.6% (2,837)	71.2% (5,622)	24.0% (1,352)	66.9% (3,759)	38.6% (2,169)	33.0% (1,854)
No qualification	19.4% (682)	28.8% (2,277)	47.4% (1,080)	87.5% (1,993)	39.8% (906)	40.3% (919)
*p-value* ^*^			<0.001	<0.001	0.222	<0.001
**Labour market status**						
In paid employment	40.6% (1,428)	37.0% (2,923)	27.0% (789)	70.4% (2,057)	37.1% (1,084)	28.6% (836)
Retired	46.2% (1,626)	48.4% (3,824)	30.0% (1,147)	73.2% (2,799)	39.0% (1,487)	38.9% (1,487)
Other	13.2% (465)	14.6% (1,152)	43.1% (496)	77.7% (896)	43.7% (504)	39.1% (450)
*p-value* ^*^			<0.001	<0.001	0.003	<0.001
**Wealth quintile**						
Wealthiest quintile	27.9% (980)	22.3% (1,765)	13.6% (240)	55.9% (988)	32.5% (575)	24.7% (436)
4^th^	23.7% (834)	20.7% (1,639)	24.4% (401)	67.8% (1,112)	39.1% (641)	30.8% (505)
3^rd^	19.2% (677)	20.4% (1,609)	34.1% (549)	76.6% (1,232)	38.5% (620)	33.4% (538)
2^nd^	17.3% (610)	19.4% (1,529)	37.5% (573)	81.4% (1,244)	41.2% (630)	39.6% (605)
Poorest quintile	11.9% (418)	17.2% (1,357)	49.3% (669)	86.7% (1,176)	44.9% (609)	50.7% (689)
*p-value* ^*^			<0.001	<0.001	<0.001	<0.001
**Self-rated general health**						
Good	75.9% (2,670)	70.0% (5,527)	26.1% (1,443)	68.9% (3,807)	36.6% (2,022)	30.1% (1,664)
Poor	24.1% (849)	30.0% (2,372)	41.7% (989)	82.0% (1,945)	44.4% (1,053)	46.7% (1,109)
*p-value* ^*^			<0.001	<0.001	<0.001	<0.001
**Limiting long-standing illness**						
No	71.9% (2,531)	68.1% (5,379)	26.8% (1,442)	69.7% (3,750)	36.8% (1,982)	30.6% (1,646)
Yes	28.1% (988)	31.9% (2,520)	39.3% (990)	79.4% (2,002)	43.4% (1,093)	44.7% (1,127)
*p-value* ^*^			<0.001	<0.001	<0.001	<0.001
**Depression (≥4 symptoms)**						
No	84.5% (2,975)	80.3% (6,342)	27.9% (1,770)	70.3% (4,461)	37.2% (2,359)	30.8% (1,957)
Yes	15.5% (544)	19.7% (1,557)	42.5% (662)	82.9% (1,291)	46.0% (716)	52.4% (816)
*p-value* ^*^			<0.001	<0.001	<0.001	<0.001
**Smoking status**						
Never-smoked	41.2% (1,451)	38.8% (3,064)	25.6% (783)	68.4% (2,097)	37.0% (1,133)	32.6% (1000)
Ex-smoker	46.4% (1,632)	46.4% (3,668)	28.7% (1,054)	72.8% (2,669)	38.5% (1,414)	34.7% (1,272)
Current smoker	12.4% (436)	14.8% (1,167)	51.0% (595)	84.4% (986)	45.2% (528)	42.9% (501)
*p-value* ^*^			<0.001	<0.001	0.002	<0.001
**Self-rated oral health**						
Good	84.2% (2,964)	82.2% (6,491)	29.1% (1,892)	71.4% (4,634)	37.0% (2,401)	32.9% (2,140)
Poor	15.8% (555)	17.8% (1,408)	38.4% (540)	79.4% (1,118)	47.9% (674)	45.0% (633)
*p-value* ^*^			<0.001	<0.001	<0.001	<0.001
**Edentulousness**						
Dentate	89.5% (3,149)	85.2% (6,735)	27.9% (1,880)	70.6% (4,753)	38.0% (2,562)	33.6% (2,262)
Edentate	10.5% (370)	14.8% (1,164)	47.4% (552)	85.8% (999)	44.0% (513)	43.9% (511)
*p-value* ^*^			<0.001	<0.001	0.006	<0.001
**OIDP**						
No impact	93.2% (3,279)	91.7% (7,246)	30.1% (2,185)	72.4% (5,246)	38.3% (2,773)	34.0% (2,467)
At least one impact	6.8% (240)	8.3% (653)	37.9% (247)	77.5% (506)	46.2% (302)	46.8% (305)
*p-value* ^*^			0.0012	0.009	0.006	<0.001

^a^Imputed n (complete case n = 887)

^b^Imputed n (complete case n = 2,329)

^c^Imputed n (complete case n = 1,392)

^d^Imputed n (complete case n = 1,155)

^*^F-test statistics for the *p-value*

Table 1 describes oral health status at follow-up by a number of baseline variables in the imputed dataset. The prevalence of poor self-rated oral health was 17.8%, 15.8% of the participants were edentate and 10.5% experienced at least one oral impact on their daily performances. ELSA participants who were not living with partner, with no qualifications, in the poorer wealth quintiles, with poor health and who were smokers were more likely to report poor oral health (*p<0*.*01*). Compared to women, men were more likely to report poor self-rated oral health although women were more likely to be edentate (*p<0*.*01*). Those aged 75+ were more likely to be edentate (*p<0*.*01*) though they did not report poorer self-rated oral health or oral impacts compared to the younger participants. Poor-self-rated oral health and edentate status were more prevalent among participants with lower structural and functional social capital, but OIDP was only associated with functional social capital.

Table 2 describes social capital at follow-up by baseline covariates in the imputed dataset. Participants aged 75+, not living with a partner, out of the labor market, in the poorer wealth quintiles, with poorer general and oral health and who were smokers were more likely to report lower structural and functional social capital (*p<0*.*01*).

Tables 3 and 4 summarize the results of the logistic regression models for the bi-directional associations between baseline social capital and self-rated oral health (Models 2 and 3 only). A detailed description and tables of results from all the models is provided in Tables C-I in S1 File.

**Table 3:** Longitudinal associations between social capital at baseline (2006–07) predicting oral health at follow-up (2010–11); (Model 2^a^ and Model 3^b^) OR (95%CI).

Social capital at baseline (2006–07)		Oral health status at follow-up (2010–11)		
		Self-rated oral health	Edentate status	OIDP
**Structural social capital**	**Models**	Poor vs Good	Edentate vs. Dentate	One oral impact vs. None
**Membership status**		OR (95%CI)	OR (95%CI)	OR (95%CI)
Not a member vs. Active member	Model 2	1.14 (0.94–1.38)	**1.49 (1.22–1.82)** ***	0.84 (0.66–1.08)
	Model 3	1.06 (0.86–1.31)	0.87 (0.58–1.30)	0.89 (0.69–1.15)
**Volunteering status**				
Not volunteering vs. Volunteering	Model 2	**1.20 (1.02–1.41** )*	**1.26 (1.05–1.51)** **	0.88 (0.72–1.07)
	Model 3	**1.26 (1.06–1.49)** *	0.99 (0.72–1.37)	0.88 (0.71–1.08)
**Functional social capital**				
**Close ties**				
Lowest tertile vs. Highest tertile	Model 2	**1.30 (1.08–1.56)** **	1.05 (0.86–1.27)	**1.44 (1.15–1.79)** **
	Model 3	**1.24 (1.02–1.51)** *	0.86 (0.60–1.25)	**1.37 (1.09–1.72)** **
**Social support**				
Lowest tertile vs. Highest tertile	Model 2	**1.41 (1.15–1.73)** **	1.13 (0.89–1.43)	**1.87 (1.43–2.44)** ***
	Model 3	**1.30 (1.04–1.62)** *	0.93 (0.60–1.45)	**1.75 (1.33–2.30)** ***

^**a**^Model adjusted for baseline demographic, socio-economic, health-related factors and smoking status (but excluding baseline dependent variable)

^b^Model adjusted for baseline demographic, socio-economic, health-related factors, smoking status, and baseline dependent variable

*p< 0.05

**p< 0.01

***p< 0.001

**Table 4:** Longitudinal associations between oral health at baseline (2006–07) predicting social capital at follow-up (2010–11); (Model 2^a^ and Model 3^b^) RRR (95%CI).

Oral health at baseline (2006–07)		Social capital at follow-up (2010–11)			
		Structural social capital		Functional social capital	
		Membership status	Volunteering status	Close ties	Social support
		No member vs. Active member	No volunteering vs. Volunteering	Lowest tertile vs. Highest tertile	Lowest tertile vs. Highest tertile
	Models	RRR (95%CI)	RRR (95%CI)	RRR (95%CI)	RRR (95%CI)
**Self-rated oral health**					
Poor vs. Good	Model 2	1.14 (0.94–1.37)	1.17 (0.99–1.38)	**1.43 (1.20–1.70)** ***	**1.47 (1.21–1.79)** ***
	Model 3	1.02 (0.80–1.30)	**1.28 (1.06–1.55)** *	**1.39 (1.14–1.70)** **	**1.36 (1.06–1.75)** *
**Edentate status**					
Edentate vs. Dentate	Model 2	**1.68 (1.33–2.12)** ***	**1.36 (1.10–1.69)** **	1.23 (0.99–1.52)	1.01 (0.80–1.28)
	Model 3	1.34 (0.99–1.81)	1.21 (0.95–1.55)	1.21 (0.94–1.55)	0.88 (0.65–1.19)
**OIDP**					
One oral impact vs. None	Model 2	0.95 (0.73–1.24)	0.93 (0.74–1.16)	1.19 (0.91–1.54)	**1.53 (1.17–2.01)** **
	Model 3	1.19 (0.86–1.66)	0.98 (0.75–1.27)	1.07 (0.80–1.43)	1.27 (0.92–1.77)

^**a**^Model adjusted for baseline demographic, socio-economic, health-related factors and smoking status (but excluding baseline dependent variable)

^b^Model adjusted for baseline demographic, socio-economic, health-related factors, smoking status, and baseline dependent variable

*p< 0.05

** *p*< 0.01

*** *p*< 0.001

### Oral health at baseline predictor of social capital at follow-up

(Table 4) In the reverse temporal association of baseline oral health predicting social capital at follow-up, ELSA respondents who had poor self-rated oral health were more likely to have fewer close ties and lower social support (Model 2). Moreover, self-rated oral health was related to change in volunteering, close ties and social support (Model 3). Respondents who experienced at least one oral impact at baseline had a higher risk of lower social support (Model 2: 1.53; 1.17–2.01), but OIDP was not related to change in social support, nor to any other measures of social capital (Model 3). Being edentate was associated with not being a member of any organization (Model 2: 1.68; 1.33–2.12) and not volunteering (Model 2: 1.36; 1.10–1.69), but was not related to change in these components of structural social capital (Model 3).

The strongest associations in Tables 3 and 4 were between social support and OIDP. The direction of the association was stronger with low social support predicting having at least one oral impact (Model 3: 1.75; 1.33–2.30) compared to oral impacts on daily performances predicting low social support (Model 3: 1.27; 0.92–1.77). Most of the Odds Ratios and Relative Risk Ratios were of similar size in each direction. However, when adjusted for the baseline values of the dependent variable, there were stronger associations from functional social capital to OIDP, rather than the reverse direction. Results from the imputed and complete case datasets (Tables J and K in S1 File) were similar, although in general, the estimates of odds ratios were smaller and the confidence intervals more precise in the imputed dataset.

## Discussion

We found evidence of bi-directional longitudinal associations between social capital and oral health. Furthermore, most of the ORs/RRRs were of similar size in each direction. However it also appears that functional social capital at baseline was a stronger predictor of oral health-related quality of life at follow-up compared to the other way around. After adjusting the models for the baseline dependent variables, the associations between functional social capital and OIDP remained stronger with social capital as a predictor of oral health rather than the other way around. Evidence of a bi-directional association also remained between self-rated oral health, volunteering and functional social capital.

The presence of such significant associations does not necessarily imply the existence of a causal association between social capital and oral health. Any observed association between social capital and oral health must be interpreted carefully.

This study showed that lack of social support at baseline was associated with an increase in poor self-rated oral health and oral impacts. This association is plausible as older adults with a partner are at risk of losing social support if their partner dies. The loss of a partner could be a major stressor which could have a negative impact on well-being and subsequently lead to depression [31], which, in turn, could affect an older person’s ability to eat food, communicate or smile.

Looking at the longitudinal associations from oral health to social capital, poor self-rated oral health at baseline was associated with lower social participation at follow-up. However, poor oral health-related quality of life was not associated with such a change in social participation. It is extremely unlikely that poor self-rated oral health can have a direct effect on social participation without going through the mechanism of one of the oral health functioning problems related to eating, smiling and socializing as assessed by the oral health quality of life measure. This suggests that poor self-rated oral health may not be a determinant of lower social participation.

Similarly, the social participation of edentate older adults could only be affected if they also reported similar problems with oral health-related quality of life. The lack of any association between OIDP and social participation suggests that the causal direction from oral health to social participation is unlikely. Thus the longitudinal association between self-rated oral health, and social participation may be caused by other unobserved confounding factors. These confounders could include changes in general health status whereby deterioration in general health could result in both perceptions of poorer oral health and lower social participation. Such health selection processes may explain some of the reported cross-sectional associations between structural social capital and oral health.

Although this study did not demonstrate any causal associations between social capital and oral health among older adults, it suggested that the direction of the association from poor oral health to lower social capital is less plausible than functional social capital as a determinant of subjective oral health. Furthermore, the change in functional social capital corresponds to plausible changes in an older person’s life course, which could result in deterioration in subjective oral health status.

The oral health measures used in this study ranged from the subjective perception of oral health (self-rated oral health), to a lifelong exposure to oral health risk factors (edentulousness). Self-rated oral health is partly influenced by oral diseases, but it also reflects current perceptions of oral health and well-being and is likely to be influenced by current exposures [19,32]. On the other hand, a person may have become edentate many years prior to the survey. This may explain the lack of association between the functional dimension of social capital and edentate status found in this study. Current levels of perceived emotional support from network members cannot influence historical life time exposures that result in the loss of teeth.

Edentulousness may have a negative impact on social life and daily activities. For example, people who are edentate may avoid participation in social activities because they are embarrassed to speak, smile, or eat in the company of other people [33]. In this study, respondents who were edentate at baseline were more likely to reduce their participation in social activities through a reduction in membership in organizations. However, it is also clear from the example above, that the pathway between edentulousness and social participation would be through oral health-related quality of life and this study found no evidence of an association between baseline oral health-related quality of life and social participation at follow-up.

This study supports previous findings that volunteering is associated with better general health and well-being [34,35]. Volunteering contributes to identifying and enhancing the role that people, especially older adults, have in the society, which in turn promotes positive psychological benefits such as increased self-esteem and happiness contributing to a positive self-evaluation of health. With respect to mental health, research shows that older adults who volunteer report fewer symptoms of depression than those who do not volunteer [35,36]. Such voluntary activities may help in the maintenance of stable socio-emotional networks as people age [37].

However there is also a clear bi-directional association between volunteering and poor self-rated oral health. This study found that poor self-rated oral health predicted a decrease in volunteering activities. So, the direction of causality between oral health and volunteering is still open to debate with the possibility that both may be confounded by other unobserved factors.

### Limitations

Although this study was the first to examine the longitudinal associations between different dimensions of social capital and oral health among older adults in a large population study using validated measures and controlling for relevant covariates, there are a few limitations. One of the limitations is that we did not have detailed clinical data on oral health such as caries, periodontal disease and number of teeth. Furthermore one of the measures of oral health, edentate status, represents the severe end of the disease spectrum. All measures of social capital and oral health are self-reported so they may be subject to common method bias. As already highlighted in the discussion, residual confounding may remain despite adjusting for known confounders. While measures of socio-economic position, general health and depression were included in the analysis, there may have been other factors that could cause the reported associations, such as negative life events, and lack of access to good quality health and social services. Oral health behavioral factors such as tooth-brushing, use of fluoride agents, sugar consumption and dental attendance are associated with oral health, but cannot cause low social capital and so are not valid confounders. Moreover, these factors were not measured in ELSA.

There were only two time-points with oral health measured, which limited the longitudinal nature of the analysis. Furthermore, the patterns of missing data in the complete cases analysis were not random, although the associations remained largely the same for both complete cases and imputed datasets. The multiple imputation analysis assumes that the data are missing at random, which may be hard to justify in ageing cohort studies. The ELSA study was designed to be representative of older adults aged 50 years and older living in England. However, for the purposes of this study, wave three of the ELSA study was used for the baseline analytical sample. Hence, they may be some selection biases associated with continuing in the ELSA study from the first wave, which limits inference to the general population of older adults in England.

### Implications for policy

In this study, lower levels of social support were associated with poorer self-rated oral health and poorer oral health-related quality of life. If these associations were causal, then, increases in social support among older adults could lead to improved oral health. However, interventions to increase the functional social capital dimension, such as social support, are difficult to implement, compared to interventions to stimulate structural social capital [38].

There are some examples of interventional programs on social capital among older adults in Japan [38–40]. Most of these interventional studies suggest some positive health effects of intergenerational voluntary activities and social participation. However, the complex social world where interventions are implemented needs to be better understood. Social capital interventions that work in particular contexts and social groups may not work in other contexts.

Given the bi-directional associations between social capital and oral health, it is also important to consider the policy implications of interventions that promote oral health among the older adults, that could, in turn, improve their social capital. Petersen and Yamamoto [41] reported that there are considerable barriers to oral health care among older people, even in industrialized countries. Older adults have less access to dental care because of their impaired mobility, the costs of dental treatment and their relatively negative attitudes to dental treatments. In Denmark, older adults were targeted by a public health program aimed at increasing their empowerment and self-care capacity-building [42]. The program resulted in an improvement in their oral health-related quality of life, oral hygiene practices and their use of dental services.

## Conclusion

Our longitudinal analysis of older adults in England has showed that social capital is linked to changes in subjective measures of oral health among older adults, rather than a cumulative life course measure of oral health such as edentulousness. Future research needs to explore the mechanisms that link social capital and oral health, and uncover the life course influences that might result in this association.

## Supporting Information

S1 File Supporting information. Text A, Missing Data. Fig A, Flowchart of the sources of missing data in the ELSA longitudinal analytical sample. Text B, Multiple imputation procedures. Table A, Characteristics of missingness (n = 5,033) to baseline sample (N = 8,552), % (n/N) distribution and OR (95%CI). Table B, Auxiliary variables predictors of missingness; OR (95%CI). Text C, Details of the models of bi-directional longitudinal associations between social capital and oral health (Imputed dataset). Table C, Logistic regression models for the association between social capital at baseline (2006–07) and poor self-rated oral health at follow-up (2010–11). Table D, Logistic regression models for the association between social capital at baseline (2006–07) and edentulousness at follow-up (2010–11). Table E, Logistic regression models for the association between social capital at baseline (2006–07) and OIDP at follow-up (2010–11). Table F, Multinomial logistic regression models for the longitudinal association between oral health at baseline (2006–07) and memberships status at follow-up (2010–11), RRR (95%CI). Table G, Binary logistic regression models for the longitudinal association between oral health at baseline (2006–07) and not volunteering at follow-up (2010–11), OR (95%CI). Table H, Multinomial logistic regression models for the longitudinal association between oral health at baseline (2006–07) and number of close ties at follow-up (2010–11), RRR (95%CI). Table I, Multinomial logistic regression models for the longitudinal association between oral health at baseline (2006–07) and social support at follow-up (2010–11), RRR (95%CI). Table J, Longitudinal associations between social capital at baseline ⟶ oral health at follow-up; and social capital at follow-up ← oral health at baseline (Model 2^a^)—complete case; OR/RRR (95%CI). Table K, Longitudinal associations between social capital at baseline ⟶ oral health at follow-up; and social capital at follow-up ← oral health at baseline (Model 3^a^)—complete case; OR/RRR (95%CI). References A.
(DOCX)
