# Supplementary material for: Is Social Capital a Determinant of Oral Health among Older Adults? Findings from the English Longitudinal Study of Ageing
Source: PLoS One. 2015 May 18;10(5):e0125557. doi: 10.1371/journal.pone.0125557 (PMC4436243; doi:10.1371/journal.pone.0125557)
Supplement: S1 File — Text A, Missing Data. Fig A, Flowchart of the sources of missing data in the ELSA longitudinal analytical sample. Text B, Multiple imputation procedures. Table A, Characteristics of missingness (n = 5,033) to baseline sample (N = 8,552), % (n/N) distribution and OR (95%CI). Table B, Auxiliary variables predictors of missingness; OR (95%CI). Text C, Details of the models of bi-directional longitudinal associations between social capital and oral health (Imputed dataset). Table C, Logistic regression models for the association between social capital at baseline (2006–07) and poor self-rated oral health at follow-up (2010–11). Table D, Logistic regression models for the association between social capital at baseline (2006–07) and edentulousness at follow-up (2010–11). Table E, Logistic regression models for the association between social capital at baseline (2006–07) and OIDP at follow-up (2010–11). Table F, Multinomial logistic regression models for the longitudinal association between oral health at baseline (2006–07) and memberships status at follow-up (2010–11), RRR (95%CI). Table G, Binary logistic regression models for the longitudinal association between oral health at baseline (2006–07) and not volunteering at follow-up (2010–11), OR (95%CI). Table H, Multinomial logistic regression models for the longitudinal association between oral health at baseline (2006–07) and number of close ties at follow-up (2010–11), RRR (95%CI). Table I, Multinomial logistic regression models for the longitudinal association between oral health at baseline (2006–07) and social support at follow-up (2010–11), RRR (95%CI). Table J, Longitudinal associations between social capital at baseline ⟶ oral health at follow-up; and social capital at follow-up ← oral health at baseline (Model 2a)—complete case; OR/RRR (95%CI). Table K, Longitudinal associations between social capital at baseline ⟶ oral health at follow-up; and social capital at follow-up ← oral health at baseline (Model 3a)—com [file pone.0125557.s001.docx]

**Supporting information**

**File S1. Supporting information**

Text A. Missing Data

Of the core wave 3 ELSA sample who had a full interview in person (N=8,552), 12.7% (n=1,089) did not return the self-completion questionnaire. A further 23.8% (n=1,776) had one or more missing values for the variables of interest. Overall, 20.8% (n=1,553) had missing information for the baseline social capital variables: 5.9% (n=439) for membership; 14.4% (n=1,071) for number of close ties and 4.5% (n=337) for social support. In addition 3.6% (n=264) had missing information for any of the covariates: 2.8% (n=213) for wealth. An additional 1,248 participants were lost to follow-up between waves 3 and 5. Out of these participants lost to follow-up, 28.5% (n=356) died in between waves; 45.4% (n= 567) refused the interviews at wave 5; 4.6% (n=58) were not well enough to participate; 2.5% (n=31); and 18.7% (n=236) for other reasons.

In the longitudinal sample of 4,439 eligible participants, 200 did not return the self-completion questionnaire at follow-up, 16.9% (n=720) had missing information on the variables of interest measured at wave 5: 4.2% (n=176) for membership; 12.1% (n=514) for number of close ties; and 3.1% (n=133) for social support (Fig A).

| ELSA sample at wave 3 (2006-07)  Full interview in person  **N=8,552 (100%)** | | |  |  |
| --- | --- | --- | --- | --- |
|  |  | | | Did not return the SCQ^a^ n=1,089 (12.2%) |
|  |  | | |  |
| **N=7,463 (87.3%)** | | |  |  |
|  |  |  |  | Total missing n=1,776 (23.8%)  Missing within SCQ^a^:  Membership n=439 (5.9%)  Number of close ties n=1,071 (14.3%)  Social support n=337 (4.5%)  Missing within CAPI^b^:  Any oral health n=2 (0.02%)  Any covariates n=264 (3.5%)  Including n=213 (2.8%) for wealth and n=44 (0.6%) for depression |
|  |  |  |  |  |
|  |  |  |  |  |
|  |  |  |  |  |
| Complete cases at wave 3  **N=5,687 (66.5%)** | | |  |  |
|  |  |  |  | Lost follow-up between waves 3 and 5  n=1,248 (21.9%) including:  Died n=356 (28.5%)  Refusals n=567 (45.4%)  Illness n=58 (4.6%)  Moved out of Britain n=31 (2.5%)  Other reasons n=236 (18.7%) |
|  |  |  |  |  |
|  |  |  |  |  |
| Follow-up sample at wave 5  **N=4,439 (51.9%)** | | |  |  |
|  |  | |  | Did not return the SCQ^a^ n=200  Additional n=720 (16.9%) missing including:  Missing within SCQ^a^:  Membership n=176 (4.1%)  Number of close ties n=514 (12.1%)  Social support n=133 (3.1%)  Missing within CAPI^b^:  Any oral health n=2 (0.04%) |
|  |  | |  |  |
|  |  | |  |  |
| Longitudinal complete cases sample  **N=3,519 (41.1%)** | | |  |  |

**Fig A Flowchart of the sources of missing data in the ELSA longitudinal analytical sample**

^a^Self-Completion Questionnaire

^b^Computer Assisting Personal Interview

Text B. Multiple imputation procedures

Multiple imputation was performed under the Missing At Random - MAR - assumption, ie, missingness in a variable is independent of the missing values themselves after conditioning on the observed data [1]. After preliminary analysis [2], the imputation model included all the variables used in the analyses models (Table A) as well as the following auxiliary variables that predict non-response in ELSA (Table B): ethnicity, marital status, housing tenure, number of people living in the household, executive function index and cognitive score. Auxiliary variables make the assumption of MAR more likely [2]. Interactions between social capital, demographic and socio-economic factors and depression were also included in the imputation model. The estimates of the logistic regression models for the 50 imputed dataset were averaged to obtain a mean estimate of the odds ratios with the standard errors adjusted according to Rubins’s rules [3].

All variables in the imputation model, including the dependent variables in the substantive analyses, were treated as multivariate responses in the imputation process. It is particularly important to include the dependent variable in the multiple imputation process as if the dependent variable is omitted from the imputation model, then the correlation between the dependent variable and any of the independent variables is assumed to be zero [4]. This assumption will bias coefficients downwards in the substantive analysis [1,4]. Thus, Young and Johnson [5] suggested that it may be acceptable for values multiply imputed on the dependent variable to be retained in the analysis. However, including the dependent variable in the imputation process results in imputed data for dead respondents, which may not be reliable. Thus, before the substantive analysis on the imputed dataset, all values imputed after a participant’s death (N=653) were removed [6].

**Table A. Characteristics of missingness (n=5,033) to baseline sample (N=8,552), % (n/N) distribution and OR (95%CI)**

| **Variables at baseline n missing/N OR (95%CI) Variables at baseline n missing/N OR (95%CI)**    **Missing % Missing %** | | | | | | | |
| --- | --- | --- | --- | --- | --- | --- | --- |
| **Demographic and socio-economic markers** | | | | | | | |
| **Age-group**  50-64  65-74  ≥75  Total | 51.6%  58.1%  76.5%  58.8% | 2,265/4,392  1,313/2,259  1,455/1,901  5,033/8,552 | 1  1.30(1.18-1.44)***  3.06(2.71-3.46)*** | **Educational status**  Some qualification  No qualification  Total | 52.4%  73.6%  58.8% | 3,127/5,964  1,903/2,585  5,030/8,549 | 1  2.53(2.29-2.80)*** |
| **Gender**  Male  Female  Total | 60.6%  57.5%  58.8% | 2,318/3,827  2,715/4,725  5,033/8,552 | 1  0.88(0.81-0.96)* | **Labour market status**  In paid employment  Retired  Others  Total | 51.8%  62.5%  62.7%  58.8% | 1,537/2,965  2,715/4,341  781/1,246  5,033/8,552 | 1  1.55(1.41-1.70)***  1.56(1.36-1.79)*** |
| **Cohabiting status**  Living with partner  No living with partner  Total | 55.7%  65.5%  58.8% | 3,224/5,790  1,809/2,762  5,033/8,552 | 1  1.51 (1.37-1.66)*** | **Wealth quintile**  Richest quintile  4^th^  3^rd^  2^nd^  Poorest quintile  Total | 44.9%  50.6%  59.6%  62.4%  72.6%  57.6% | 798/1,778  856/1,690  1,000/1,677  1,013/1,623  1,110/1,528  4,777/8,296 | 1  1.26(1.10-1.44)**  1.81(1.58-2.08)***  2.04(1.78-2.34)***  3.26(2.82-3.77)*** |
| **Health related factors** | | | | | | | |
| **Self-rated health**  Good  Poor  Total | 53.9%  69.1%  58.8% | 3,128/5,798  1,902/2,751  5,030/8,549 | 1  1.91(1.73-2.10)*** | **Depression**  No  Yes  Total | 55.8%  68.7%  58.5% | 3,760/6,735  1,197/1,741  4,957/8,476 | 1  1.74(1.56-1.95)*** |
| **Long-standing illness**  No  Yes  Total | 55.1%  66.0%  58.8% | 3,104/5,635  1,922/2,910  5,026/8,545 | 1  1.59(1.44-1.74)*** | **Smoking status**  Never smoked  Ex-smoker  Current smoker  Total | 55.3%  59.4%  66.0%  58.8% | 1,792/3,243  2,393/4,025  846/1,282  5,031/8,550 | 1  1.19(1.08-1.30)***  1.57(1.37-1.80)*** |
| **Oral health status** | | | | | | | |
| **Self-rated oral health**  Good  Poor  Total | 57.6%  64.3%  58.8% | 4,032/6,996  998/1,553  5,030/8,549 | 1  1.32(1.18-1.48)*** | **OIDP^a^**  No impact  At least one impact  Total | 58.1%  66.8%  58.8% | 4,549/7,828  483/723  5,032/8,551 | 1  1.45(1.23-1.70)*** |
| **Edentulousness**  Dentate  Edentate  Total | 55.8%  74.1%  58.8% | 3,972/7,121  1,060/1,430  5,032/8,551 | 1  2.27(2.00-2.58)*** |  |  |  |  |
| **Social capital indicators** | | | | | | | |
| **Membership status**  Active member  Passive member  No member  Total | 42.4%  50.8%  57.8%  49.9% | 1,007/2,376  1,364/2,687  1,134/1,961  3,505/7,024 | 1  1.40(1.25-1.57)***  1.86(1.65-2.10)*** | **Number of close ties**  Highest tertile  Middle tertile  Lowest tertile  Total | 43.4%  42.8%  48.0%  44.9% | 894/2,060  819/1,914  1,160/2,418  2,873/6,392 | 1  0.97(0.86-1.10)  1.20(1.07-1.35)** |
| **Volunteering status**  Volunteering  No volunteering  Total | 47.0%  63.1%  58.8% | 1,064/2,262  3,967/6,288  5,031/8,550 | 1  1.92(1.75-2.12)*** | **Social support**  Highest tertile  Middle tertile  Lowest tertile  Total | 48.8%  49.7%  52.9%  50.6% | 1,040/2,133  1,174/2,361  1,393/2,632  3,607/7,126 | 1  1.04(0.92-1.17)  1.18(1.05-1.32)** |

^a^Oral Impacts on Daily Performances

**p<0.05; **p<0.01; ***p<0.001*

**Table B. Auxiliary variables predictors of missingness; OR (95%CI)**

| **Variables OR (95%CI) Variables OR (95%CI)** | | | | | | | |
| --- | --- | --- | --- | --- | --- | --- | --- |
| **Memory Function Index**  <mean score  >mean score |  |  | 1  0.43(0.39-0.47)*** | **Marital status**  Single  Married  Divorced  Widowed |  |  | 1  0.96(0.80-1.15)  1.25(1.01-1.56)*  1.69(1.37-2.09)*** |
| **Executive Function Index**  <mean score  >mean score |  |  | 1  0.46(0.42-0.50)*** | **HSE source survey**  1998  1999  2001  2002  2003  2004 |  |  | 1  1.08(0.95-1.23)  1.00(0.90-1.10)  1.31(0.97-1.78)  0.83(0.68-1.03)  1.30(0.96-1.75) |
| **Ethnicity**  White  No white  **Housing tenure**  Own  Mortgage  Rent |  |  | 1  2.55(1.85-3.51)***  1  0.90(0.81-1.00)  2.11(1.86-2.40)*** | **Household type**  Single  Lone  Couple  Extended family |  |  | 1  1.00(0.78-1.28)  0.67(0.61-0.74)***  0.94(0.75-1.18) |
|  | | | | | | | |

Text C. Details of the models of bi-directional longitudinal associations between social capital and oral health (Imputed dataset)

Social capital at baseline predictor of oral health at follow-up

*Social capital at* baseline *predictor of self-rated oral health at follow-up (Table C)*

Baseline social capital (both structural and functional components) was statistically related to self-rated oral health in the age-adjusted model (Model 1). Not being a member of any organization (1.55; 1.30-1.84), not volunteering 1.58 (1.36-1.83), having less than 6 closes ties 1.48 (1.24-1.77) and having the lowest perceived social support 1.85 (1.57-2.18) were all associated with poor self-rated oral health at follow-up. Adjusting for baseline demographic, socio-economic, health and behavioral factors (Model 2) resulted in a decrease in the odds ratios for all social capital measures. While the findings were still significant for volunteering, close ties and social support, the association between membership and self-rated oral health was no longer statistically significant (1.14; 0.94-1.38).

A more detailed analysis suggested that wealth, education, depression, and self-rated general health contributed the most to the reduction in the odds ratios. After adjusting for baseline self-rated oral health (Model 3), volunteering, fewer close ties and low social support at baseline were all related to change in self-rated oral health at follow-up.

**Table C. Logistic regression models for the association between social capital**

**at baseline (2006-07) and poor self-rated oral health at follow-up (2010-11)**

|  | Model 1 | Model 2 | Model 3 |
| --- | --- | --- | --- |
| **Social capital at baseline (2006-07)** | OR (95% CI) | OR (95% CI) | OR (95% CI) |
| **Membership status**  Active member  Passive member  Not a member | 1  1.17 (0.98-1.39)  1.55 (1.30-1.84)*** | 1  1.09 (0.91-1.30)  1.14 (0.94-1.38) | 1  1.06 (0.87-1.28)  1.06 (0.86-1.31) |
| **Volunteering status**  Volunteering  Not volunteering | 1  1.58 (1.36-1.83)*** | 1  1.20 (1.02-1.41)* | 1  1.26 (1.06-1.49)* |
| **Number of close ties**  Highest tertile  Middle tertile  Lowest tertile | 1  1.14 (0.94-1.38)  1.48 (1.24-1.77)*** | 1  1.14 (0.93-1.39)  1.30 (1.08-1.56)** | 1  1.12 (0.91-1.39)  1.24 (1.02-1.51)* |
| **Social support**  Highest tertile  Middle tertile  Lowest tertile | 1  1.21 (1.02-1.43)*  1.85 (1.57-2.18)*** | 1  1.09 (0.91-1.30)  1.41 (1.15-1.73)** | 1  1.05 (0.87-1.27)  1.30 (1.04-1.62)* |

Model 1: Age adjusted

Model 2: Model 1 + demographic, socio-economic, health and smoking status at baseline

Model 3: Model 2 + self-rated oral health at baseline

**p* < 0.05; ***p* < 0.01; ****p* < 0.001

N=7,899

*Social capital at baseline predictor of edentulousness at follow-up (Table D)*

Lower social capital in all aspects but close ties, was associated to edentate status in the age adjusted models (Model 1). In Model 2, for all social capital explanatory variables measured at baseline, only not being a member of any organization and not volunteering were still significantly associated with edentate status at follow-up (1.49; 1.22-1.82 and 1.26; 1.05-1.51, respectively). Among the socio-economic factors, wealth and education contributed the most to weaken the association, and smoking status. When baseline edentate status was adjusted for (Model 3), none of the baseline social capital measures were associated with edentate status at follow-up.

**Table D. Logistic regression models for the association between social capital**

**at baseline (2006-07) and edentulousness at follow-up (2010-11)**

|  | Model 1 | Model 2 | Model 3 |
| --- | --- | --- | --- |
| **Social capital at baseline (2006-07)** | OR (95% CI) | OR (95% CI) | OR (95% CI) |
| **Membership status**  Active member  Passive member  Not a member | 1  1.37 (1.13-1.65)**  2.59 (2.15-3.10)*** | 1  1.12 (0.92-1.36)  1.49 (1.22-1.82)*** | 1  0.87 (0.61-1.25)  0.87 (0.58-1.30) |
| **Volunteering status**  Volunteering  Not volunteering | 1  1.98 (1.68-2.34)*** | 1  1.26 (1.05-1.51)** | 1  0.99 (0.72-1.37) |
| **Number of close ties**  Highest tertile  Middle tertile  Lowest tertile | 1  0.94 (0.77-1.14)  1.17 (0.98-1.40) | 1  0.92 (0.75-1.13)  1.05 (0.86-1.27) | 1  0.89 (0.59-1.34)  0.86 (0.60-1.25) |
| **Social support**  Highest tertile  Middle tertile  Lowest tertile | 1  1.22 (1.02-1.46)*  1.48 (1.24-1.76)*** | 1  1.10 (0.89-1.34)  1.13 (0.89-1.43) | 1  1.11 (0.78-1.58)  0.93 (0.60-1.45) |

Model 1: Age adjusted

Model 2: Model 1 + demographic, socio-economic, health and smoking status at baseline

Model 3: Model 2 + edentate status at baseline

**p* < 0.05; ***p* < 0.01; ****p* < 0.001

N=7,899

*Social capital at baseline predictor of Oral Impacts on Daily Performances (OIDP) at follow-up (Table E****)***

Neither membership, nor volunteering at baseline were significantly associated with OIDP at follow-up (Model 1). On the other hand, fewer close ties and lower social support were significantly associated with OIDP. For example, having fewer than 6 close ties at baseline was associated with increased odds of experiencing an oral impact at follow-up (1.61; 1.30-1.99) compared to having more than 9 close ties at baseline.

Similarly, those respondents with the lowest tertile of social support were 2.24 (1.78-2.81) times more likely to report at least one oral impact when compared to those respondents who reported a high level of social support. Moreover, these associations remained relatively strong even after the adjustment for demographic, socio-economic, health and behavioral factors.

In order to estimate the influence of the social capital exposures on change in oral health-related quality of life, baseline OIDP variable was added to the regression model (Model 3). The odds ratios for lowest tertile of close ties (1.37; 1.09-1.72) and social support (1.75; 1.33-2.30) were reduced but the associations remained significant.

**Table E. Logistic regression models for the association between social capital**

**at baseline (2006-07) and OIDP at follow-up (2010-11)**

|  | Model 1 | Model 2 | Model 3 |
| --- | --- | --- | --- |
| **Social capital at baseline (2006-07)** | OR (95% CI) | OR (95% CI) | OR (95% CI) |
| **Membership status**  Active member  Passive member  Not a member | 1  0.93 (0.76-1.14)  1.16 (0.93-1.45) | 1  0.86 (0.69-1.06)  0.84 (0.66-1.08) | 1  0.87 (0.69-1.08)  0.89 (0.69-1.15) |
| **Volunteering status**  Volunteering  Not volunteering | 1  1.14 (0.95-1.38) | 1  0.88 (0.72-1.07) | 1  0.88 (0.71-1.08) |
| **Number of close ties**  Highest tertile  Middle tertile  Lowest tertile | 1  1.24 (0.97-1.57)  1.61 (1.30-1.99)*** | 1  1.24 (0.97-1.58)  1.44 (1.15-1.79)** | 1  1.22 (0.95-1.56)  1.37 (1.09-1.72)** |
| **Social support**  Highest tertile  Middle tertile  Lowest tertile | 1  1.52 (1.21-1.91)**  2.24 (1.78-2.81)*** | 1  1.42 (1.12-1.81)**  1.87 (1.43-2.44)*** | 1  1.37 (1.07-1.75)*  1.75 (1.33-2.30)*** |

Model 1: Age adjusted

Model 2: Model 1 + demographic, socio-economic, health and smoking status at baseline

Model 3: Model 2 + OIDP at baseline

**p* < 0.05; ***p* < 0.01; ****p* < 0.001

N=7,899

Oral health at baseline predictors of social capital at follow-up

*Oral health at baseline predictors of membership status at follow-up (Table F)*

In the age adjusted models (Model 1), respondents who reported poor self-rated oral health and at least one oral impact on daily performance were more likely not to be members of any organization compared to those respondents who reported good self-rated oral health and did not experience any OIDP (1.73; 1.47-2.04 and 1.51; 1.20-1.91, respectively). A stronger association was found for edentate status. Compared to respondents who were dentate, edentate respondents were 3.12 (2.52-3.86) times more likely not to be members of any organization.

In Model 2, the estimates for the risk of no membership in comparison to active membership reduced and the associations between self-rated oral health, oral impacts and membership were fully explained (1.14; 0.94-1.37 and 0.95; 0.73-1.24, respectively). On the other hand, the risk of no membership versus active membership was 1.68 (1.33-2.12) higher among edentate respondents. Furthermore, once the model was adjusted for the baseline membership status (Model 3), the association between edentate status and membership was marginally not significant (1.34; 0.99-1.81). In other words, being edentate at baseline marginally reduced the chances social participation at follow-up.

**Table F. Multinomial logistic regression models for the longitudinal association between oral health**

**at baseline (2006-07) and memberships status at follow-up (2010-11), RRR (95%CI)**

|  | **Memberships status at follow-up (2010-11)** | | | | |  |  |
| --- | --- | --- | --- | --- | --- | --- | --- |
|  | Model 1 Model 2 Model 3 | Model 2 | | Model 3 | |  |  |
| **Oral health at baseline (2006-07)** | Not a member vs  active member RRR (95% CI) | | Not a member vs  active member RRR (95% CI) | | Not a member vs  active member RRR (95% CI) | |  |
| **Self-rated oral health**  Good  Poor  **Edentulousness**  Dentate  Edentate  **OIDP**  No impact  At least 1 impact | 1  1.73 (1.47-2.04)***  1  3.12 (2.52-3.86)***  1  1.51 (1.20-1.91)*** | | 1  1.14 (0.94-1.37)  1  1.68 (1.33-2.12)***  1  0.95 (0.73-1.24) | | 1  1.02 (0.80-1.30)  1  1.34 (0.99-1.81)  1  1.19 (0.86-1.66) | |  |

Model 1: contains the age adjusted association between oral health measures at baseline (separately) and membership status at follow-up

Model 2: contains model 1 adjusted for demographic, socio-economic, health, and smoking status at baseline

Model 3: contains model 2 adjusted for membership status at baseline

* *p* < 0.05; ** *p* < 0.01; *** *p* < 0.001

N = 7,899

*Oral health at baseline predictors of volunteering status at follow-up (Table G)*

The age-adjusted longitudinal model (Model 1) revealed that reporting poor self-rated oral health, being edentate and having experienced at least one oral impacts at baseline were associated with not being a volunteer at follow-up. For instance, the odds of not being a volunteer at follow-up was 2.20 (1.80-2.69) times greater among the respondents who were edentate compared to the dentate at baseline.

Taking account of the effect of baseline covariates on the association between baseline oral health measures and volunteering at follow-up, neither self-rated oral health, nor oral impacts at baseline were associated to volunteering at follow-up (1.17; 0.99-1.38 and 0.93; 0.74-1.16, respectively). Noting that, however, the association between self-rated oral health and volunteering was marginally significant. In the autoregressive model, which adjusted for baseline volunteering status (Model 3), a significant association was found between self-rated oral health and volunteering. Self-rated oral health at baseline was related to change in volunteering across waves 3 and 5 (1.28; 1.06-1.55).

**Table G. Binary logistic regression models for the longitudinal association between oral health**

**at baseline (2006-07) and not volunteering at follow-up (2010-11), OR (95%CI)**

|  | **Not volunteering at follow-up (2010-11)** | | | | |  |  |
| --- | --- | --- | --- | --- | --- | --- | --- |
|  | Model 1 Model 2 Model 3 | Model 2 | | Model 3 | |  |  |
| **Oral health at baseline (2006-07)** | OR (95% CI) | | OR (95% CI) | | OR (95% CI) | |  |
| **Self-rated oral health**  Good  Poor  **Edentulousness**  Dentate  Edentate  **OIDP**  No impact  At least 1 impact | 1  1.59 (1.36-1.84)***  1  2.20 (1.80-2.69)***  1  1.30 (1.06-1.60)* | | 1  1.17 (0.99-1.38)  1  1.36 (1.10-1.69)**  1  0.93 (0.74-1.16) | | 1  1.28 (1.06-1.55)*  1  1.21 (0.95-1.55)  1  0.98 (0.75-1.27) | |  |

Model 1: contains the age-adjusted association between oral health measures at baseline (separately) and volunteering status at follow-up

Model 2: contains model 1 adjusted for demographic, socio-economic, health, and smoking status at baseline

Model 3: contains model 2 adjusted for volunteering status at baseline

* *p*< 0.05; ** *p*< 0.01; *** *p*< 0.001

N = 7,899

*Oral health at baseline predictors of number of close ties at follow-up (Table H)*

In the age-adjusted model (Model 1), significant associations were found between all indicators of oral health and the lowest tertile of close ties. This suggests that poor oral health at baseline might reduce the number of close relationships among older adults at follow-up. For example, compared to those respondents who reported good self-rated oral health at baseline, respondents who reported poor self-rated oral health were more likely to have fewer than 7 close ties at follow-up (1.65; 1.39-1.96).

Even after adjusting for baseline covariates (Model 2) the association remained significant for self-rated oral health (1.43; 1.20-1.70), while edentate status and OIDP at baseline were no longer significantly associated with close ties at follow-up.

Model 3 adjusted for baseline number of close ties, and examined the effect of self-rated oral health on change in the number of close ties. Compared to respondents who reported good self-rated oral health, those who reported poor self-rated oral health were more likely to have fewer than 7 close ties (1.39; 1.14-1.70).

**Table H. Multinomial logistic regression models for the longitudinal association between oral health**

**at baseline (2006-07) and number of close ties at follow-up (2010-11), RRR (95%CI)**

|  | **Number of close ties at follow-up (2010-11)** | | | | |  |  |
| --- | --- | --- | --- | --- | --- | --- | --- |
|  | Model 1 Model 2 Model 3 | Model 2 | | Model 3 | |  |  |
| **Oral health at baseline (2006-07)** | Lowest tertile vs  highest tertile RRR (95% CI) | | Lowest tertile vs  highest tertile RRR (95% CI) | | Lowest tertile vs  highest tertile RRR (95% CI)) | |  |
| **Self-rated oral health**  Good  Poor  **Edentulousness**  Dentate  Edentate  **OIDP**  No impact  At least 1 impact | 1  1.65 (1.39-1.96)***  1  1.34 (1.09-1.66)**  1  1.41 (1.10-1.81)** | | 1  1.43 (1.20-1.70)***  1  1.23 (0.99-1.52)  1  1.19 (0.91-1.54) | | 1  1.39 (1.14-1.70)**  1  1.21 (0.94-1.55)  1  1.07 (0.80-1.43) | |  |

Model 1: contains the age-adjusted association between oral health measures at wave 3 (separately) and number of close ties at follow-up

Model 2: contains model 1 adjusted for demographic, socio-economic, health, and smoking status at baseline

Model 3: contains model 2 adjusted for number of close ties at baseline

* *p* < 0.05; ** *p* < 0.01; *** *p* < 0.001

N = 7,899

*Oral health at baseline predictors of social support at follow-up (Table I)*

Model 1 shows that respondents with poor self-rated oral health, who were edentate and had reported at least one oral impact at baseline were more likely to have lower social support at follow-up. Respondents with poor self-rated oral health had a higher risk of having lower social support than those who reported good self-rated oral health (2.01; 1.70-2.37). A slightly stronger association was found for OIDP. The risk of being in the lowest tertile of social support versus the highest tertile was 2.09 (1.66-2.64) times greater in respondents who had an oral impact than those who had no oral impact.

The estimates of the association between the most subjective measures of oral health (self-rated oral health and OIDP) and social support were substantially reduced after adjusting for the baseline covariates but the associations were still statistically significant (Model 2). On the other hand the association between edentate and social support was explained after adjustment for baseline covariates.

In Model 3, after accounting for baseline social support, only the association between self-rated oral health and social support persisted (1.36; 1.06-1.75).

**Table I. Multinomial logistic regression models for the longitudinal association between oral health**

**at baseline (2006-07) and social support at follow-up (2010-11), RRR (95%CI)**

|  | **Social support at follow-up (2010-11)** | | | | |  |  |
| --- | --- | --- | --- | --- | --- | --- | --- |
|  | Model 1 Model 2 Model 3 | Model 2 | | Model 3 | |  |  |
| **Oral health at baseline (2006-07)** | Lowest tertile vs  highest tertile RRR (95% CI) | | Lowest tertile vs  highest tertile RRR (95% CI) | | Lowest tertile vs  highest tertile RRR (95% CI) | |  |
| **Self-rated oral health**  Good  Poor  **Edentulousness**  Dentate  Edentate  **OIDP**  No impact  At least 1 impact | 1  2.01 (1.70-2.37)***  1  1.38 (1.14-1.67)**  1  2.09 (1.66-2.64)*** | | 1  1.47 (1.21-1.79)***  1  1.01 (0.80-1.28)  1  1.53 (1.17-2.01)** | | 1  1.36 (1.06-1.75)*  1  0.88 (0.65-1.19)  1  1.27 (0.92-1.77) | |  |

Model 1: contains the age-adjusted association between oral health measures at baseline (separately) and social support at follow-up

Model 2: contains model 1 adjusted for demographic, socio-economic, health, and smoking status at baseline

Model 3: contains model 2 adjusted for social support at baseline

* *p* < 0.05; ** *p* < 0.01; *** *p* < 0.001

N = 7,899

**Table J. Longitudinal associations between social capital at baseline ⟶ oral health at follow-up;**

**and social capital at follow-up** ← **oral health at baseline (Model 2^a^) – complete case;**

**OR/RRR (95%CI)**

|  | **Poor self-rated oral health** | **Edentate status** | **OIDP** |
| --- | --- | --- | --- |
|  | Model 2  OR/RRR (95%CI) | Model 2  OR/RRR (95%CI) | Model 2  OR/RRR (95%CI) |
| ***Structural social capital***  **Membership status**  Not a member vs.  Active member | ⟶  1.15 (0.89-1.48)  ←  1.17 (0.90-1.51) | ⟶  **1.59 (1.18-2.13)****  ←  **1.71 (1.25-2.35)**** | ⟶  0.90 (0.66-1.22)  ←  0.98 (0.68-1.40) |
| **Volunteering status**  Not volunteering vs.  Volunteer | ⟶  1.10 (0.89-1.36)  ←  **1.25 (1.01-1.56)*** | ⟶  **1.34 (1.02-1.75)***  ←  **1.38 (1.04-1.83)*** | ⟶  0.88 (0.68-1.13)  ←  1.00 (0.74-1.36) |
| ***Functional social capital***  **Close ties**  Lowest tertile | ⟶  **1.28 (1.01-1.61)***  ←  **1.50 (1.19-1.91)**** | ⟶  1.05 (0.80-1.37)  ←  1.19 (0.89-1.57) | ⟶  **1.51 (1.13-2.00)****  ←  **1.41 (1.01-1.97)*** |
| **Social support**  Lowest tertile | ⟶  **1.48 (1.13-1.95)****  ←  **1.53 (1.17-2.02)**** | ⟶  1.09 (0.78-1.52)  ←  1.15 (0.82-1.62) | ⟶  **2.26 (1.61-3.18)*****  ←  **2.06 (1.39-3.05)***** |

⟶ Association between social capital at wave 3 and oral health at wave 5

← Association between oral health at wave 3 and social capital at wave 5

**^a^**Model adjusted for baseline covariates (but excluding baseline dependent variable)

**p*< 0.05; ***p*< 0.01; ****p*< 0.001

**Table K. Longitudinal associations between social capital at baseline ⟶ oral health at follow-up;**

**and social capital at follow-up** ← **oral health at baseline (Model 3^a^) – complete case;**

**OR/RRR (95%CI)**

|  | **Poor self-rated oral health** | **Edentate status** | **OIDP** |
| --- | --- | --- | --- |
|  | Model 3  OR/RRR (95%CI) | Model 3  OR/RRR (95%CI) | Model 3  OR/RRR (95%CI) |
| ***Structural social capital***  **Membership status**  Not a member vs.  Active member | ⟶  1.04 (0.79-1.36)  ←  0.95 (0.74-1.22) | ⟶  0.89 (0.51-1.56)  ←  1.32 (0.90-1.94) | ⟶  0.95 (0.69-1.31)  ←  1.17 (0.76-1.81) |
| **Volunteering status**  Not volunteering vs.  Volunteer | ⟶  1.16 (0.92-1.46)  ←  **1.41 (1.10-1.81)**** | ⟶  1.08 (0.68-1.73)  ←  1.22 (0.89-1.70) | ⟶  0.90 (0.69-1.17)  ←  1.11 (0.77-1.59) |
| ***Functional social capital***  **Close ties**  Lowest tertile vs.  Highest tertile | ⟶  1.18 (0.92-1.51)  ←  **1.47 (1.12-1.93)**** | ⟶  0.85 (0.52-1.39)  ←  1.15 (0.82-1.60) | ⟶  **1.45 (1.08-1.95)***  ←  1.34 (0.91-1.98) |
| **Social support**  Lowest tertile vs.  Highest tertile | ⟶  1.26 (0.94-1.70)  ←  1.19 (0.84-1.67) | ⟶  1.03 (0.56-1.89  ←  1.10 (0.72-1.66) | ⟶  **2.03 (1.43-2.88)*****  ←  1.51 (0.94-2.45) |

⟶ Association between social capital at wave 3 and oral health at wave 5

← Association between oral health at wave 3 and social capital at wave 5

^a^Model adjusted for baseline covariates and baseline dependent variable

**p*< 0.05; ** *p*< 0.01; *** *p*< 0.001

**References A**

1. Little RJA, Rubin DBS (2002) Statistical Analysis with Missing Data New York: John Wiley & Sons, Inc.

2. Spratt M, Carpenter J, Sterne JA, Carlin JB, Heron J, et al. (2010) Strategies for multiple imputation in longitudinal studies. Am J Epidemiol 172: 478-487.

3. Rubin DB (1987) Multiple imputation for non-response in surveys. New York: John Wiley.

4. Graham JW (2008) Missing Data Analysis: Making It Work in the Real World. Annual Review of Psychology 60: 549-576.

5. Young R, Johnson DR (2010) Imputing the Missing Y's:Implications for Survey Producers and Survey Users in Proceedings of the AAPOR Conference Abstracts 6242–6248.

6. Harel O, Hofer SM, Hoffman L, Pedersen NL, Johansson B (2007) Population Inference with Mortality and Attrition in Longitudinal Studies on Aging: A Two-Stage Multiple Imputation Method. Experimental Aging Research 33: 187-203.
